# Monocarboxylate Transporter 1 (MCT1) is an independent prognostic biomarker in endometrial cancer

**DOI:** 10.1186/s12907-017-0067-7

**Published:** 2017-12-28

**Authors:** Ayşe Latif, Amy L. Chadwick, Sarah J. Kitson, Hannah J. Gregson, Vanitha N. Sivalingam, James Bolton, Rhona J. McVey, Stephen A. Roberts, Kay M. Marshall, Kaye J. Williams, Ian J. Stratford, Emma J. Crosbie

**Affiliations:** 1 Division of Pharmacy and Optometry, Faculty of Biology, Medicine and Health, University of Manchester, Manchester, UK; 2 Gynaecological Oncology Research Group, Division of Cancer Sciences, Faculty of Biology, Medicine and Health, University of Manchester, Level 5 – Research, St Mary’s Hospital, Oxford Road, Manchester, M13 9WL UK; 3 Department of Histopathology, Central Manchester University Hospitals NHS Foundation Trust, Manchester Academic Health Science Centre, Manchester, UK; 4 Division of Population Health, Health Services Research and Primary Care, Faculty of Biology, Medicine and Health, University of Manchester, Manchester, UK; 5 Department of Obstetrics and Gynaecology, St Mary’s Hospital, Central Manchester University Hospitals NHS Foundation Trust, Manchester Academic Health Science Centre, Manchester, UK

**Keywords:** Endometrial cancer, Monocarboxylate transporters, Hypoxia, Glycolysis

## Abstract

**Background:**

Endometrial cancer (EC) is a major health concern due to its rising incidence. Whilst early stage disease is generally cured by surgery, advanced EC has a poor prognosis with limited treatment options. Altered energy metabolism is a hallmark of malignancy. Cancer cells drive tumour growth through aerobic glycolysis and must export lactate to maintain intracellular pH. The aim of this study was to evaluate the expression of the lactate/proton monocarboxylate transporters MCT1 and MCT4 and their chaperone CD147 in EC, with the ultimate aim of directing future drug development.

**Methods:**

MCT1, MCT4 and CD147 expression was examined using immunohistochemical analysis in 90 endometrial tumours and correlated with clinico-pathological characteristics and survival outcomes.

**Results:**

MCT1 and MCT4 expression was observed in the cytoplasm, the plasma membrane or both locations. CD147 was detected in the plasma membrane and associated with MCT1 (*p* = 0.003) but not with MCT4 (*p* = 0.207) expression. High MCT1 expression was associated with reduced overall survival (*p* = 0.029) and remained statistically significant after adjustment for survival covariates (*p* = 0.017).

**Conclusion:**

Our data suggest that MCT1 expression is an important marker of poor prognosis in EC. MCT1 inhibition may have potential as a treatment for advanced or recurrent EC.

**Electronic supplementary material:**

The online version of this article (10.1186/s12907-017-0067-7) contains supplementary material, which is available to authorized users.

## Background

In 2012, it is estimated that more than 30,000 new diagnoses of endometrial cancer (EC) were made worldwide. In the UK, EC is the fourth most common cancer in women, with more than 9000 new cases diagnosed in 2013 [1]. EC usually presents at an early stage following the onset of postmenopausal bleeding and is generally cured by hysterectomy and bilateral salpingo-oophorectomy. However, surgery can be dangerous for obese and elderly women, with significant risk of anaesthetic and surgical complications. Mainstay treatment for advanced EC includes cytotoxic chemotherapy and/or hormonal therapy. However, women with advanced, recurrent or metastatic disease have a poor prognosis with only 7-12 months median survival [2–4]. There is therefore a clear unmet clinical need for new therapies to modify disease outcome.

The rising incidence of EC over the last three decades has paralleled the obesity epidemic [1]. Clinical and epidemiological studies have shown that women with obesity and type II diabetes have an increased risk of EC [5–7]. In obese and diabetic women, tissues involved in insulin-mediated glucose uptake (such as the liver) become insulin resistant, leading to hyperglycaemia and hyperinsulinaemia [8]. The increased risk of EC is further influenced by high levels of circulating glucose acting as an energy source and contributing to metabolic adaptations in rapidly proliferating tumour cells. Most malignancies have been shown to utilise aerobic glycolysis as the predominant energy pathway (known as “the Warburg effect”) [9]. As a consequence, large amounts of lactic acid are produced, which must be exported out of cells by monocarboxylate transporters (MCTs), to avoid acid-induced apoptosis.

MCTs are transmembrane proteins encoded by the SLC16A family of genes. Among all 14 MCT family members, four MCTs (MCT1-MCT4) are characterized as lactate/proton symporters. In particular, MCT4 (efflux of lactate) and MCT1 (both influx and efflux of lactate) are among the most important regulators of intracellular pH homeostasis in tumours and other high glycolytic tissues [10, 11]. Upregulation of MCTs has been shown in many malignancies including colorectal [12, 13], cervix [14], breast [15, 16], prostate [17], central nervous system [18, 19], soft tissue [20], gastrointestinal [21, 22], oral cavity [23], urothelial [24] and lung carcinomas [25] and high expression is associated with poor prognosis. Both MCT1 and 4 require association with the ancillary protein CD147 (also known as EMMPRIN or Basigin) for plasma membrane expression and activity [26–28]. CD147 is a pleiotropic plasma membrane glycoprotein that stimulates the synthesis of several matrix metalloproteinases, thus promoting tumour invasion [29–31]. Maturation and cell surface expression of CD147 is also dependent on MCT1 or MCT4 expression [32]. CD147 is upregulated [31] and linked to poor prognosis in malignancies of various origins, including endometrium [33].

The potential role of MCTs in tumour metabolism has promoted their emergence as new targets for cancer therapy. Despite evidence that MCT1, MCT4 and CD147 are poor prognostic factors in several cancer types, their significance in EC is not known. The aim of this study was to analyse the expression of MCT1, MCT4 and CD147 in EC and relate these findings to clinico-pathological features and survival outcomes.

## Methods

### Case selection

Ethical approval for this study was obtained from NRES Committee London - Fulham (reference 12/LO/0364). It was conducted in accordance with the conditions and principles outlined in the EU Directive 2001/20/EC and Good Clinical Practice, including the Data Protection Act 1998. Tumour tissues were obtained from 90 sequential EC patients who underwent hysterectomy (between 2011 and 2013) and donated their tumours for future research at St Mary’s Hospital, Manchester, UK. All participants provided written, informed consent for their clinical data and tumour samples to be stored anonymously and used for future research. Their paraffin-embedded tumour specimens were retrieved from the pathology archives and cut into 4-μm serial sections for immunohistochemical analysis. The median age of the cohort was 67 years (IQR range 57.7-74 years) and there were 47 endometrioid (EEC) and 43 non-endometrioid (Non-EEC) tumours. The non-EEC group comprised tumours of carcinosarcoma (*n* = 11), clear-cell (*n* = 9), serous (*n* = 6), mixed (*n* = 16) and undifferentiated (n = 1) histology. Mixed tumours were clear cell/high grade endometrioid (*n* = 13), clear cell/ high grade serous (*n* = 2) or high grade serous/ endometrioid (n = 1). Clinico-pathological data were obtained from patients’ hospital medical records, original pathology results and death certificates, where appropriate, including age at diagnosis, Body Mass Index (BMI), tumour histological type, grade, Federation of Gynecology and Obstetrics (FIGO) 2009 stage, tumour size, lymphovascular space involvement (LVSI), depth of myometrial invasion, last follow-up date, date of recurrence, type of recurrence, death and cause of death (Table 1). The average follow up time was 32.4 months and there were 28 recurrences and 22 deaths, of which 12 were EC-specific.

**Table 1:** Expression of MCT1, MCT4 and CD147 in EC tumours and their correlation with clinico–pathological characteristics

		MCT1 (*N* = 89)			MCT4 (*N* = 87)			CD147 (N = 87)		
	*N* = 90 (%)	Low (<200) *N* = 48 (53.9%)	High (≥200) *N* = 41 (46.1%)	*p*	Low (<200) *N* = 59 (67.8%)	High (≥200) *N* = 28 (32.2%)	*p*	Low (<200) *N* = 31 (35.6%)	High (≥200) *N* = 56 (64.4%)	*p*
Age of Onset (Median, IQR years)	67 (57.7–74)	69 (58.2–74.7)	67 (56–73)	0.917	67 (56–74)	69.5 (61.5–75.2)	0.760	67 (61–73)	68 (56–75)	0.836
BMI (Median)	29.4 (26–37.8)	29 (26.1–35.1)	39 (26–39.5)	0.764	30.7 (26–39.4)	28 (26.2–34.9)	0.596	28.2 (23.2–34.9)	30.2 (26.3–39.5)	0.557
Diabetic, N (%)				0.094			0.949			0.678
Yes (0)	71 (78.9%)	41 (58.6%)	29 (41.4%)		46 (67.6%)	22 (32.4%)		25 (36.8%)	43 (63.2%)	
No (1)	19 (21.1%)	7 (36.8%)	12 (63.2%)		13 (68.4%)	6 (31.6%)		6 (31.6%)	13 (68.4%)	
Grade, N (%)				0.218			0.313			0.737
I/II	42 (46.7%)	25 (60.9%)	16 (39.1%)		30 (73.1%)	11 (26.9%)		15 (37.5%)	25 (62.5%)	
III	48 (53.3%)	23 (47.9%)	25 (52.1%)		29 (63.1%)	17 (36.9%)		16 (34.1%)	31 (65.9%)	
Histological Type, N (%)				0.614			0.409			0.988
Endometrioid (1)	47 (52.2%)	26 (56.5%)	20 (43.5%)		26 (56.5%)	20 (43.5%)		16 (35.6%)	29 (64.4%)	
^a^Non–endometrioid (2)	43 (47.8%)	22 (51.2%)	21 (48.8%)		22 (51.2%)	21 (48.8%)		15 (35.7%)	27 (64.3%)	
FIGO 2009 Stage, N (%)				0.752			0.988			0.026
I	58 (64.4%)	31 (54.4%)	26 (45.6%)		37 (66.1%)	19 (33.9%)		25 (44.6%)	31 (55.4%)	
II	11 (12.2%)	5 (45.5%)	6 (54.5%)		9 (81.8%	2 (18.2%)		2 (18.2%)	9 (81.8%)	
III	19 (21.1%)	10 (52.6%)	9 (47.4%)		12 (66.7%)	6 (33.3%)		4 (22.2%)	14 (77.8%)	
IV	2 (2.2%)	2 (100%)	0 (0%)		1 (50.0%)	1 (50.0%)		0 (0%)	2 (100%)	
Tumour Size, N (%)				0.234			0.866			0.117
Less than 2 cm (1)	9 (10.0%)	5 (55.6%)	4 (44.4%)		7 (77.8%)	2 (22.2%)		4 (50.0%)	4 (50.0%)	
2–5 cm (2)	46 (51.1%)	28 (62.2%)	17 (37.8%)		32 (71.1%)	13 (28.9%)		19 (43.2%)	25 (56.8%)	
Bigger than 5 cm (3)	11 (26.7%)	10 (41.7%)	14 (58.3%)		16 (72.7%)	6 (27.3%)		6 (25.0%)	18 (75.0%)	
Missing	24 (26.7%)									
LVSI, N (%)				0.928			0.692			0.948
No (0)	50 (55.6%)	26 (53.1%)	23 (46.6%)		33 (67.3%)	16 (32.7%)		17 (35.4%)	31 (64.6%)	
Yes (1)	37 (41.1%)	20 (54.1%)	17 (45.9%)		25 (71.4%)	10 (28.6%)		13 (36.1%)	23 (63.9%)	
Missing	3 (3.3%)									
Depth of Myometrial Invasion, N (%)				0.680			0.203			0.512
Less than (<) 50%	50 (55.6%)	26 (52.0%)	24 (48.0%)		36 (73.5%)	13 (26.5%)		16 (32.7%)	33 (67.3%)	
More than or equal to (≥) 50%	40 (44.4%)	22 (56.4%)	17 (43.6%)		23 (60.5%)	15 (39.5%)		15 (39.5%)	23 (60.5%)	

^a^Non-EEC tumours are composed of tumours with carcinosarcoma, mixed, clear cell, serous and undifferentiated histology

### Immunohistochemistry (IHC)

All IHC was performed using a fully automated IHC platform Leica BOND-MAX together with Bond™ Polymer Refine Detection kit (DS9800) and on-board retrieval system. The detection kit is based on a biotin-free, polymeric horseradish peroxidase (HRP)-linker antibody conjugate system for the detection of tissue-bound mouse and rabbit IgG and some mouse IgM primary antibodies. It is intended for staining sections of formalin-fixed, paraffin-embedded tissue on the Bond™ automated system. The sections were quenched using hydrogen peroxide and subjected to MCT1 (Santa Cruz, sc-365,501, 1:100 dilution), MCT4 (Santa Cruz, sc-376,140, 1:100 dilution) and CD147 (Fitzgerald Industries International, Clone UM-8D6, 1:100 dilution) primary antibody according to standard validated Protocol F written by Leica. Negative (isotype) controls were used for quality assurance. Following addition of post primary reagents the primary antibody was refined and visualized using the substrate chromogen, 3,3′-Diaminobenzidine tetrahydrochloride hydrate (DAB) via a brown precipitate. Tissue sections were counterstained with haematoxylin to visualize cell nuclei and were permanently mounted.

### Immunohistochemical evaluation

Immunoreaction in sections was evaluated for whole cell staining in whole tumour sections. A semi-quantitative scoring system was devised to take into account differences in staining intensity and distribution in different tumours. The observed staining intensities were given a score 0–3, from zero intensity staining (0) to high intensity staining (3), each staining intensity was multiplied by the percentage of tumour cells staining positively at each intensity. This gave a score range of 0-300. The tumours were separated into two groups as ‘low expression <200’ and ‘high expression ≥200’. Immunohistochemical evaluation was performed by two blinded independent observers and discrepancies were settled by consensus to determine the final score.

### Statistical analysis

All data was stored and analysed using SPSS statistical software (version 22). Comparisons between staining positivity and clinico-pathological characteristics were made using Chi- square (χ^2^), Fishers exact test (as appropriate) or Mann Whitney U test (for continuous variables). Pearson correlation coefficients were calculated to assess the association between metabolic markers and/or clinico-pathological characteristics. Overall survival (OS), cancer specific survival (CSS) and recurrence-free survival (RFS) were analysed using Kaplan-Meier curves and compared using the Log-rank (Mantel-Cox) tests. Analyses adjusted for previously identified prognostic factors were performed using Cox proportional hazards models using a large covariate set (grade, FIGO 2009 stage, tumour size, LVSI and depth of myometrial invasion). Further, given the low event number a simpler model adjusted for grade and stage was also fitted. As CSS and RFS endpoints had fewer events a model which just adjusted for grade as a stratification variable was used for these endpoints. In all statistical analyses threshold for significance was *p* < 0.05.

## Results

### MCT1, MCT4 and CD147 expression, distribution and subcellular localization in EC

**Fig. 1:**
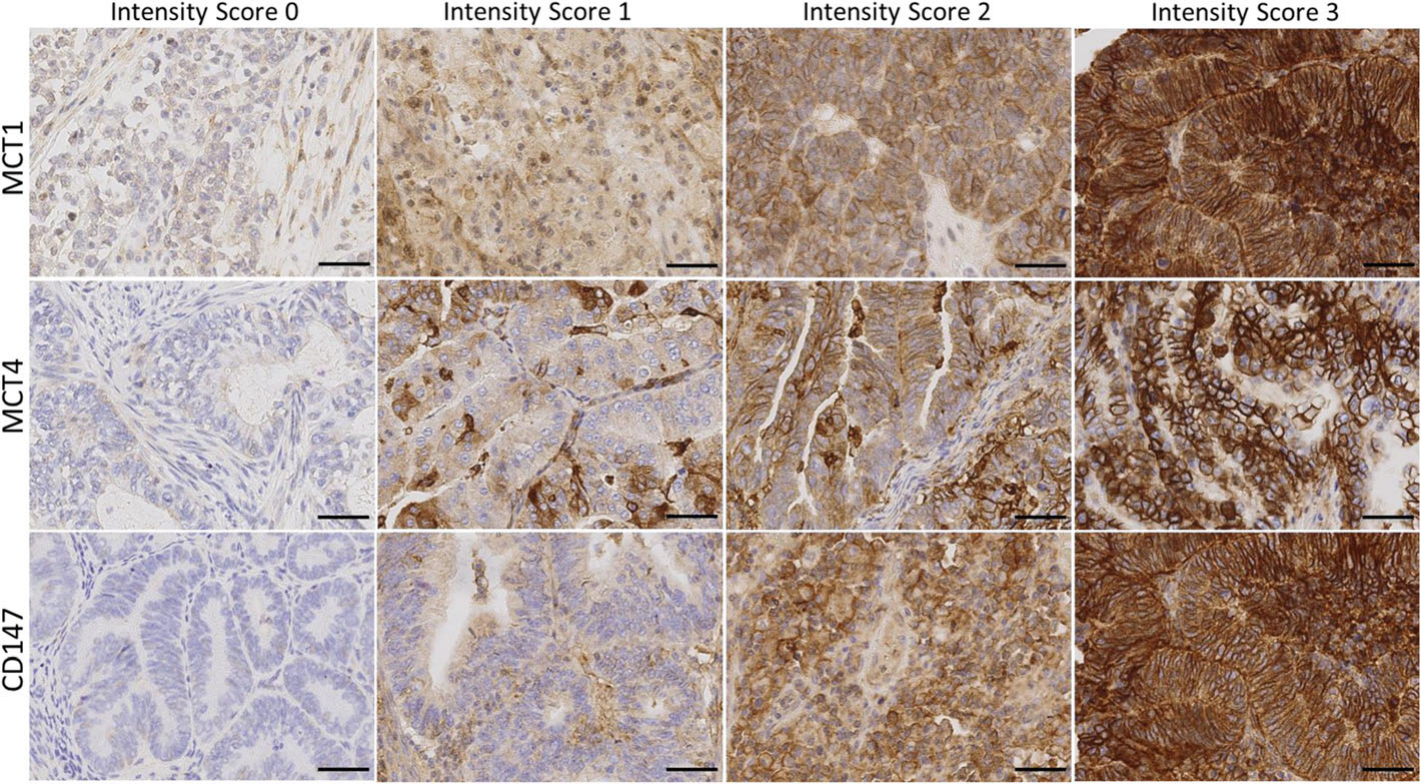
Representative immunohistochemical reactions for intensity score 0, 1, 2 and 3 for MCT1, MCT4 and CD147 expression in non-EEC tumours. The expression of MCT1 was localized to the cytoplasm, the plasma membrane or both locations. MCT4 and CD147 expression was always observed in the plasma membrane with some level of cytoplasmic staining. Scale bars represent 50 μm

In this study, immunohistochemical evaluation of MCT1, MCT4 and CD147 was performed in 90 EC (47 endometrioid [EEC] and 43 non-endometrioid [non-EEC]) tumours. All aforementioned markers were expressed at varying levels. Representative images of tumour sections stained for MCT1, MCT4 and CD147 are shown in Fig. 1. MCT1 was expressed in the cytoplasm, the plasma membrane or in both locations. MCT4 and CD147 were always expressed in the plasma membrane accompanied by some degree of cytoplasmic staining (Fig. 1).

**Fig. 2:**
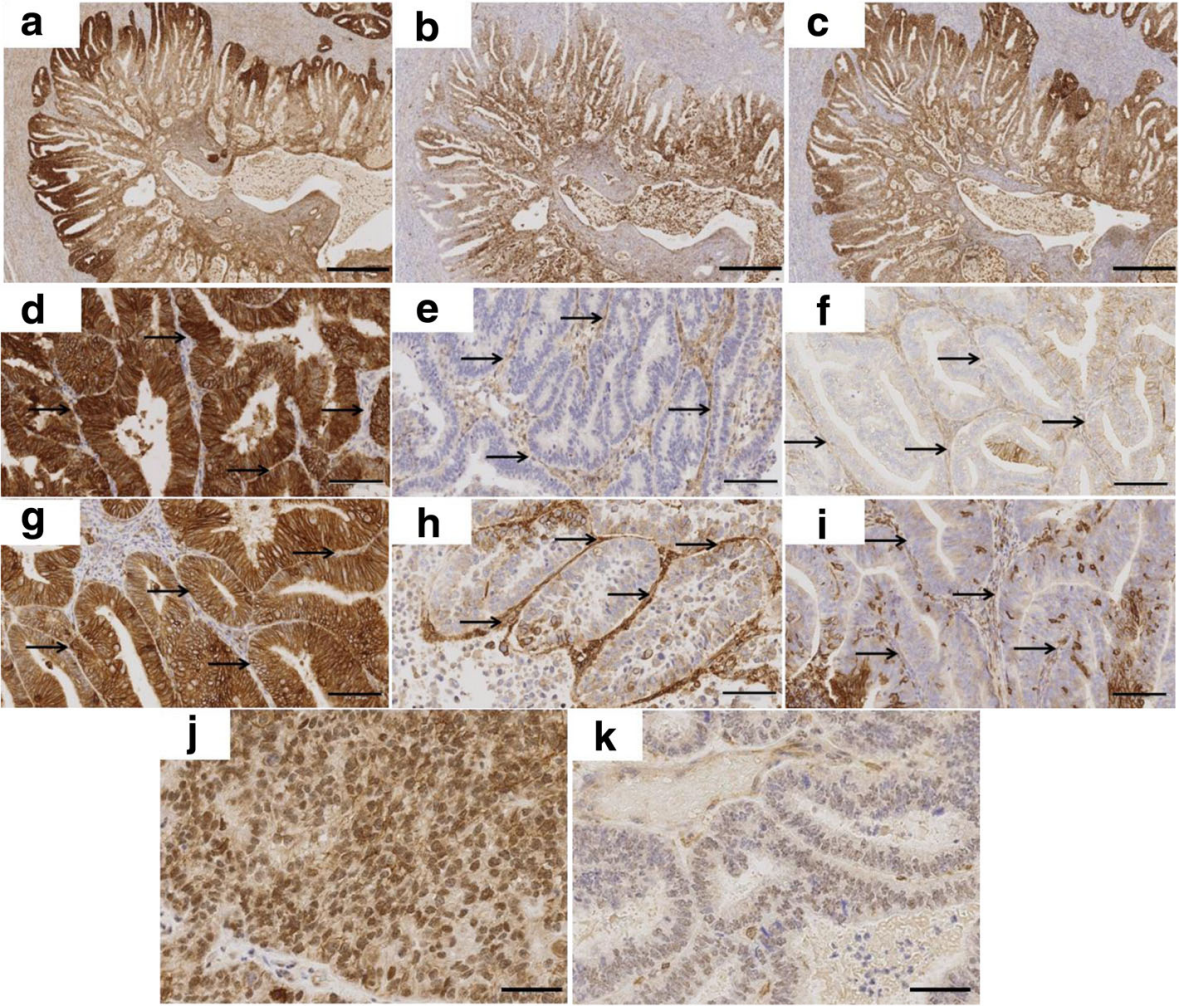
Representative examples of MCT1, MCT4 and CD147 distribution and subcellular localization in EC tumours. **a** MCT1 and **b** MCT4 were found to dominate different zones of the same tumour, peripheral and central zones, respectively. **c** CD147 expression was observed in the same zone with MCT1. In some, strong glandular staining for **d** MCT1 and **g** MCT4 was observed with no stromal staining. In others, stromal expression of **e** MCT1 and **h** MCT4 were higher than glandular staining. Some tumours showed similar levels of **f** MCT1 and **i** MCT4 expression in both glandular and stromal tumour compartments. In more than half of the tumours, nuclear MCT1 staining (**j** and **k**) was observed. Arrows indicate stromal compartment between malignant glands. Scale bars represent 500 μm, 100 μm and 50 μm in (**a**-**c**), (**d**-**i**) and (**j**-**k**), respectively

MCT1 and MCT4 staining patterns were highly variable both within and between tumours. MCT1 was generally observed in peripheral zones (Fig. 2a) whereas MCT4 was expressed in central zones of the same tumour (Fig. 2b). Strong CD147 expression was mainly observed in those areas that were heavily stained with MCT1 (Fig. 2c). In contrast, in areas with strong MCT4 expression CD147 expression was weaker (Fig. 2c). Stromal and myometrial expression of MCT1 and MCT4 was also seen (Fig. 2d-i). In some, stromal and glandular staining displayed high contrast; malignant glands showed strong MCT1 expression whilst stromal staining was negative (or mild) or vice versa. In others, MCT1 and MCT4 expression was similar across stromal and glandular tumour compartments. Although the nucleus is not a usual location for MCT1 (based on current knowledge of its function), nuclear MCT1 expression was present in more than half of the tumours (present 58.9%, absent 40.1%; Fig. 2j-k).

There was a wide range in the intensity and distribution of staining, which was scored as described in the methods section. Briefly, tumours were recorded as high expressers when their score was equal to or exceeded 200; tumours with scores below this were regarded as low expressers. Interestingly, for MCT1, using the score of 200 resulted in 53.9% of tumours scoring low and 46.1% of tumours scoring high. Using the same 200 score cut off high MCT4 expression was observed in 32.2% of tumours and high levels of CD147 expression were observed in 64.4% of the tumours. There was a significant association between expression of CD147 and MCT1 (*p* = 0.003) but not MCT4 (*p* = 0.207) in our cohort (Pearson correlation coefficients = 0.32 and 0.14, respectively). There was no significant association between MCT1 and MCT4 expression (*p* = 0.93).

### Associations between metabolic markers and clinico-pathological data

There were no significant associations between the clinico-pathological characteristics of the tumours and expression levels of either MCT1 or MCT4 (Table 1). CD147 expression was significantly associated with FIGO 2009 stage (Pearson Correlation Coefficient = 0.24), although this association would not be considered significant if allowance was made for the number of characteristics tested.

### MCT1 expression is associated with reduced overall survival in EC

**Fig. 3:**
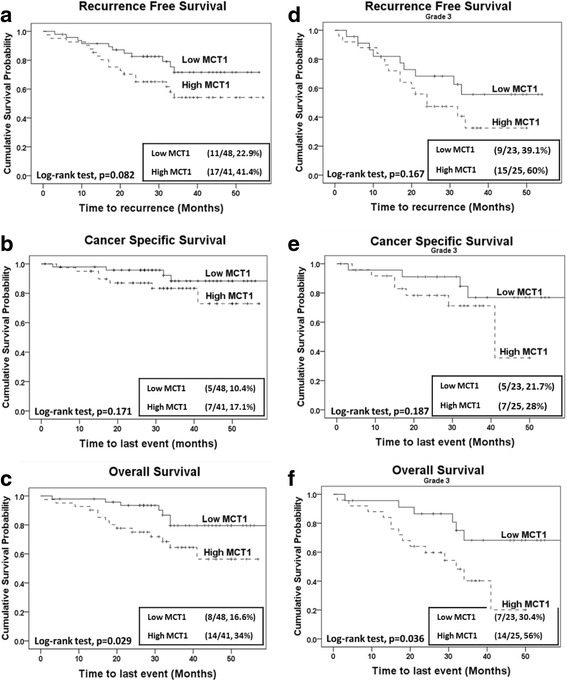
Kaplan-Meier survival analysis for MCT1 expression using unadjusted and grade stratified models. Recurrence-free, cancer specific and overall survival is shown for unadjusted model in (**a**, **b** and **c**)**;** in grade III tumours (from grade stratified model) only (**d**, **e** and **f**), respectively. High MCT1 expression significantly reduced overall survival in **c** unadjusted and **f** in grade III tumours. The events for each arm are shown in insets (event/total, %)

When the expression of these three metabolic markers was correlated with patient survival parameters (recurrence free, cancer specific and overall survival), MCT1 was identified as a prognostic marker in EC. Using an unadjusted Log-rank test, patients with high MCT1 expression showed reduced recurrence free and cancer specific survival and a significantly reduced overall survival (Fig. 3a-c, respectively).

Amongst survival outcome covariates (grade, FIGO stage, tumour size, LVSI and depth of myometrial invasion), grade and FIGO 2009 stage were the most significant prognostic factors in our study (Table 2). In order to evaluate whether MCT1 expression was an independent prognostic factor, MCT1 expression was tested in a large adjusted model with all of the listed covariates and a simpler model with just the major predictors, grade and stage (Table 2). In these analyses, MCT1 remained statistically significant after adjustment for all six possible covariates as well as in the simpler stage and grade-adjusted model (Table 2).

**Table 2:** Unadjusted and adjusted Cox proportional hazard analysis for overall survival

Overall survival									
	Unadjusted model Overall cohort			Large adjusted model Overall cohort			Simple adjusted model Overall cohort		
	HR	95% CI	*p*	HR	95% CI	*p*	HR	95% CI	*p*
MCT1	**1.622**	**1.030–2.554**	**0.037**	**2.123**	**1.145–3.937**	**0.017**	**1.963**	**1.199–3.214**	**0.007**
Grade	**4.684**	**1.715–12.79**	**0.003**	**3.210**	**1.136–9.076**	**0.028**	**3.797**	**1.372–10.50**	**0.010**
Stage	**2.288**	**1.500–3.489**	**<0.001**	**1.965**	**1.078–3.579**	**0.027**	**2.080**	**1.300–3.328**	**0.002**
Size	1.269	0.585–2.750	0.547	0.681	0.306–1.515	0.346	**–**	**–**	**–**
LVSI	2.347	0.935–5.891	0.069	1.185	0.378–3.718	0.771	**–**	**–**	**–**
MI	1.447	0.609–3.436	0.403	1.121	0.340–3.690	0.851	**–**	**–**	**–**

Grade categorised as Grade I/II and III tumours; Stage: FIGO 2009 stage categorised as 1, 2, 3 and 4; Size categorised as <2 cm, between 2 and 5 cm and bigger than 5 cm; LVSI: Lymphovascular space involvement categorised as “Yes” and “No”; MI: Depth of myometrial invasion categorised as <50% and more than 50%; HR: hazard ratio, CI: confidence interval. Bold text indicates statistically significant data at *p* < 0.05 level

Due to the limited number of events observed for recurrence free and cancer specific survival, full adjustment for covariates was not possible for these endpoints. Therefore, we used a simpler adjustment for the most important predictor of survival in our cohort, which was grade, and fitted this as a stratified Log-rank test and Cox regression model.

When the overall cohort was stratified according to grade (I/II vs III), similar to the unadjusted model, an increased trend towards reduced recurrence free, cancer specific and overall survival was observed in patients with high MCT1 expressing grade III (mainly composed of Non-EEC) tumours (Fig. 3d-f). This effect reached statistical significance for overall survival (Table 3). There were too few events in patients with grade I/II tumours for meaningful statistical analysis (only composed of EEC tumours, shown in Additional file 1: Figure S1). Grade stratified Cox proportional hazard analysis performed on other markers (CD147 and MCT4) showed an increased risk of earlier time to event for patients with high CD147 expression but this effect did not reach statistical significance. There were no significant associations between these two metabolic markers and any of the survival parameters evaluated in this study (Table 3).

**Table 3:** Cox proportional hazard analysis of recurrence free, cancer specific and overall survival in unadjusted and grade stratified model

		Unadjusted Model			Grade Stratified Model		
		HR	95% CI	*p*	HR	95% CI	*P*
MCT1	RFS	1.390	0.951–2.031	0.089	1.302	0.889–1.907	0.175
	CSS	1.518	0.820–2.809	0.184	1.508	0.805–2.826	0.199
	OS	**1.622**	**1.030–2.554**	**0.037**	**1.604**	**1.012–2.541**	**0.044**
MCT4	RFS	0.829	0.539–1.275	0.394	0.771	0.501–1.186	0.237
	CSS	0.685	0.318–1.474	0.333	0.641	0.279–1.381	0.256
	OS	0.905	0.563–1.453	0.679	0.846	0.526–1.359	0.489
CD147	RFS	1.309	0.853–2.008	0.217	1.331	0.867–2.044	0.191
	CSS	1.251	0.644–2.431	0.508	1.383	0.704–2.718	0.347
	OS	1.358	0.822–2.245	0.232	1.469	0.884–2.443	0.138

*RFS* recurrence free survival, *OS* overall survival, *CSS* cancer specific survival, *HR* hazard ratio, *CI* confidence interval. Bold text indicates statistically significant data at *p* < 0.05 level

## Discussion

This is the first study to evaluate the prognostic significance of MCT1, MCT4 and CD147 expression in EC. Using both unadjusted and adjusted analyses we found high MCT1 expression to be an independent factor predicting poor survival in patients with EC. This finding is consistent with previous studies examining the role of MCT1 in other cancer types [14, 20, 22, 34] and supports its development as a therapeutic target in EC and other malignancies.

Increased glucose uptake, glycolysis and adaptation to acidosis are key events during cancer progression [35]. MCT1 and MCT4 are important contributors to the regulation of tumour intracellular pH and induction of extracellular acidosis. Understanding the role of these transporters in tumours will clarify their contribution to tumour metabolism and the malignant phenotype. Recent efforts have been made to identify the prognostic significance of MCT1 [14, 15, 21, 23, 24, 34] and MCT4 (reviewed by [36]) in different tumour types, however none have studied their role in EC.

In EC, MCT1 and MCT4 were expressed in the cytoplasm, the plasma membrane or both. The observed cytoplasmic as well as membranous MCT1 and MCT4 staining suggests either the presence of alternative mechanisms that ensure acid efflux and maintenance of intracellular pH or the use of non-glycolytic metabolic pathways in EC. Interestingly, mitochondrial membrane expression of MCT1 [37] and MCT4 [13, 38–40] has been described in other tumour types. Moreover, an increased cytoplasmic (as well as plasma membrane) expression of MCT1 is reported in basal like breast cancers [15] suggesting it may have additional functions such as transportation of lactate/pyruvate through the mitochondrial membrane. Further, in more than half of the tumours evaluated in this study, nuclear MCT1 expression was present. This is consistent with a previous study of soft tissue sarcomas [20]. To the best of our knowledge, this is the first study showing expression of nuclear MCT1 in EC. As the cellular localization does not fit with the classic role of this protein as a transmembrane transporter, this finding suggests an additional, not yet described, role for MCT1. We found no statistically significant association between nuclear MCT1 expression and recurrence free, overall or cancer-specific survival.

Based on their functional differences, both MCT1 and MCT4 display tissue specific patterns of distribution. MCT1 and MCT4 are variably expressed in tumours originating from the breast, colon, lung and ovary (reviewed by [41]). In addition, different MCTs are known to transport lactic acid between different cell types within the same tumour tissue. It has been proposed that hypoxic and glycolytic tumour cells distant from functional blood vessels use MCT4 to export lactic acid, which is then absorbed by the peripheral oxidative tumour cells through MCT1 [42]. Indeed, in some of the tumours studied here, MCT1 and MCT4 were expressed independently in different epithelial zones of the same tumour, suggesting that metabolic needs varied according to the precise location of cells expressing different transporters. This observation is consistent with the metabolic heterogeneity described by Sonveaux et al. [42] and in a recent study of urothelial bladder cancer [43]. Moreover, differential expression of MCT1 and MCT4 were observed in the stromal compartment of these tumours. A meta-analysis performed by Bovenzi et al. [36], showed that MCT4 expression in the tumour stromal compartment was associated with reduced overall and disease free survival, however, there was not sufficient information to perform a similar analysis on MCT1. Nevertheless, this finding suggests that the differential stromal expression of MCT1 and 4 observed in our EC cohort might be due to a metabolic symbiosis established between the tumour and its microenvironment supporting highly proliferative epithelial cancer cells [44].

CD147 plays an important role in cancer progression [29, 30] and the regulation of MCT1 and MCT4 activity and expression [45]. In this study, we observed CD147 to be primarily associated with the plasma membrane and often co-localizing with MCT1. This association was not seen with MCT4 expression. This is consistent with other cancer types such as ovarian and oral cavity tumours [23, 40] and provides evidence for the importance of CD147 in MCT1 localization and function in EC. Lack of significant association between membrane localization of MCT4 and CD147 leads us to speculate that MCT4 plasma localization in EC may depend on an additional protein such as CD44 [40, 46].

## Conclusions

In summary, this is the first study evaluating the expression of MCT1, MCT4 and CD147 in EC. The results demonstrate MCT1 to be an important marker for overall survival in EC. The differential staining patterns for MCT1 in low grade EEC and non-EEC tumours indicate metabolic differences between the tumour types. Our data suggest that agents targeting MCT1 may have potential in the treatment of this disease and support the notion that exploitation of metabolic targets may pave the way for personalised EC prevention and therapy.

## Additional file

Additional file 1: Figure S1. Kaplan-Meier survival analysis for grade I/II endometrial tumours. (PDF 309 kb)

## Abbreviations

BMI: Body mass index
CSS: Cancer-specific survival
DAB: Diaminobenzidine tetrahydrochloride
EC: Endometrial cancer
EEC: Endometrioid endometrial cancer
FIGO: Federation of Gynecology and Obstetrics
HRP: Horseradish peroxidase
Ig: Immunoglobulin
IHC: Immunohistochemistry
IQR: Interquartile range
LVSI: Lymphovascular space involvement
MCT: Monocarboxylate transporter
OS: Overall survival
RFS: Recurrence free survival
