# Supplementary material for: Monocarboxylate Transporter 1 (MCT1) is an independent prognostic biomarker in endometrial cancer
Source: BMC Clin Pathol. 2017 Dec 28;17:27. doi: 10.1186/s12907-017-0067-7 (PMC5745908; doi:10.1186/s12907-017-0067-7)
Supplement: Additional file 1: Figure S1. — Kaplan-Meier survival analysis for grade I/II endometrial tumours. (PDF 309 kb) [file 12907_2017_67_MOESM1_ESM.pdf]

Monocarboxylate Transporter 1 (MCT1) is an independent prognostic biomarker in endometrial cancer

Ayşe Latif[1], Amy L. Chadwick[1,2], Sarah J. Kitson[2], Hannah J. Gregson[2], Vanitha N. Sivalingam[2], James Bolton[3], Rhona J. McVey[3], Stephen A. Roberts[4], Kay M. Marshall[1], Kaye J. Williams[1], Ian J. Stratford[1], Emma J. Crosbie[2,5*]

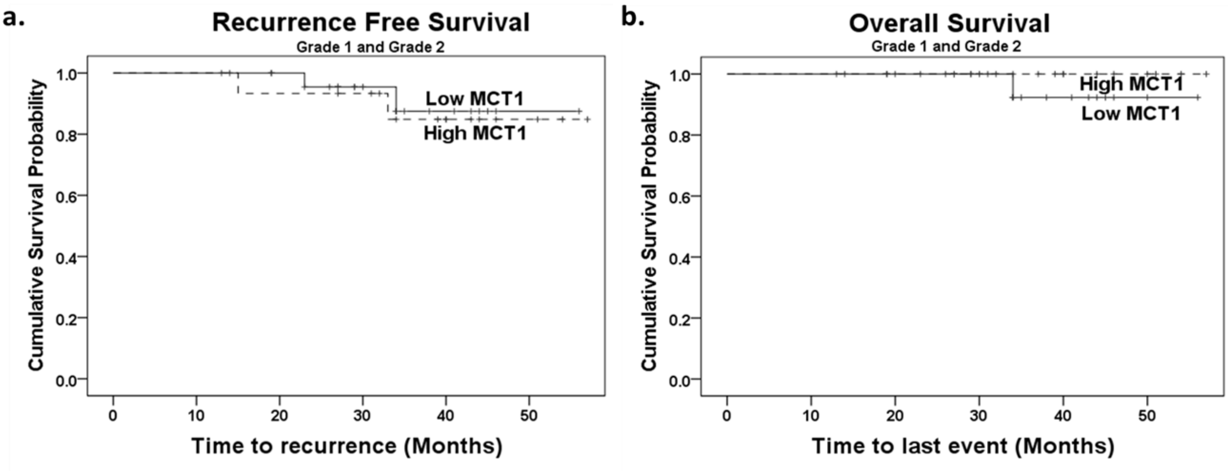

Supplementary Figure 1: Kaplan-Meier survival analysis for grade I/II tumours. In grade I/II tumours **(a)** Recurrence free survival; there were two recurrences in each of the low and high MCT1 expressing tissues. **(b)** Overall survival; there was only one death in the low MCT1 tumours and non in the high MCT1 expressing tumours. As there were no events in grade I/II tumours no plot can be drawn for cancer specific survival.
